# Supplementary material for: Genomic Divergence Shaped the Genetic Regulation of Meiotic Homologous Recombination in Brassica Allopolyploids
Source: Mol Biol Evol. 2025 Apr 2;42(4):msaf073. doi: 10.1093/molbev/msaf073 (PMC11982612; doi:10.1093/molbev/msaf073)
Supplement: msaf073_Supplementary_Data [file msaf073_supplementary_data.zip › Supporting_Information (3).pdf]

Supporting Information

Fig. S1 Recombination rates in Centimorgan (cM) for each of the 10 homologous A chromosomes in hybrids carrying (A) *B. oleracea* C_o or (B) *B. napus* C_n chromosomes. Statistical differences, determined by a Bonferroni-corrected Chi-squared test (5% threshold), are indicated with letters (a–c).

Fig. S2 Frequency of crossovers per chromatid for the progenies derived from hybrids carrying *B. oleracea* C_o chromosomes.

Fig. S3 Relative recombination rate across 10 bins along each individual A chromosome and for the 10 A chromosomes pooled in each hybrid. KL divergence: divergence between the observed crossover landscape and the theoretical flat landscape (dashed line). *** indicates p-values<10^{-3} for testing H0: 'flat landscape' (10^4 bootstraps). KL comparison tests: pairwise comparisons between above-mentioned KL values. p-values for testing H0: 'KL values are identical' are obtained by 10^4 bootstraps.

Fig. S4 Landscape flatness for each individual A chromosome and for the 10 A chromosomes pooled between the hybrids carrying *B. oleracea* C_o or *B. napus* C_n chromosomes. Flatness is proxied by the KL divergence between the observed crossover landscape (distribution across 10 bins) and the corresponding theoretical flat distribution.

Fig. S5 Relationship between the relative recombination rates (normalized per A chromosome, %) and their relative distance from the centromeres (%) in hybrids carrying (A) *B. oleracea* C_o or (B) *B. napus* C_n chromosomes. $A_rA_{r'}$ (purple circles): y=0.1637x-1.4544; R^2=0.55. $A_rA_{r'}+1C_{o(9)}$ (black diamonds): y=0.0630x+3.3110; R^2=0.19. $A_rA_{r'}C_o$ (light purple squares): y=0.01348x+5.6515; R^2=n.s.. $A_nA_{r'}$ (blue circles): y=0.1387x-0.2705; R^2=0.54. $A_nA_{r'}+1C_{n(9)}$ (black diamonds): y=0.1086x+1.1521; R^2=0.34. $A_nA_{r'}C_n$ (light blue squares): y=0.0446x+4.1816; R^2=0.11.

Fig. S6 Density of distances between successive crossovers across 10 bins (relative to chromosome length) for each individual A chromosome and for the 10 A chromosomes pooled in each hybrid. KL divergence: divergence between the experimental distribution and the expected one in the absence of interference (obtained by shuffling crossovers across different individuals in the population; dashed line). *** indicates p-values<10^{-3} for testing H0:

'no interference' (10^4 bootstraps). KL comparison tests: pairwise comparisons between above-mentioned KL values. p-values for testing H0: 'KL values are identical' are obtained by 10^4 bootstraps.

Fig. S7 Divergence from the non-interference situation for each individual A chromosome and for the 10 A chromosomes pooled between hybrids carrying *B. oleracea* C_o or *B. napus* C_n chromosomes. Values of KL divergence between the observed distribution of inter-crossover distances across 10 bins and the corresponding distribution without interference (obtained by shuffling the data). Statistical differences, determined by a Bonferroni-corrected test using 10^4 bootstraps (5% threshold), are indicated with letters (a, b, c).

Fig. S8 Meiotic observations of hybrids carrying a diploid $A_nA_{r'}$ genome and the additional chromosomes C04, C08 and C09 of *B. napus* single or in combination. (**1-7.A**) Pollen Mother Cells showing ten bivalents as well as one (**1-3.A**), two (**4-6.A**) or three (**7.A**) univalents indicated by the red stars. FISH analyses carried out with the BAC Bob014O06, revealing the C chromosomes unpaired (**1-7.B**, in green), the BAC KBrH033J07 showing the A05 and C04 homoeologous pairs (**1-7.C**, in red), the BAC KBrB043F18 showing the A09 and C08 homoeologous pairs (**1-7.D**, in purple), and the BAC KBrH080A08 showing the A10 and C09 homoeologous pairs (**1-7.E**, in orange). Bars, 5 µm.

Fig. S9 Frequency of crossovers per chromatid for the progenies derived from hybrids carrying *B. napus* C_n chromosomes.

Fig. S10 Recombination rates in Centimorgan (cM) for each of the 10 homologous A chromosomes in hybrids carrying additional *B. napus* C_n chromosomes.

Table S1. Characteristics of SNP markers genetically mapped on the 10 homologous A chromosomes of the *B. rapa* cv. 'Chiifu-401' genome sequence (Wang *et al*., 2011), used for hybrids carrying *B. oleracea* C_o and *B. napus* C_n chromosomes.

Table S2. Landscape flatness analysis. Detail of the KL values and p-values corresponding to the results presented in Figures 3 and Supplementary Figures S3 and S4. The numbers 1, 2, 3 indicate the hybrids of the columns Pop_1, Pop_2 and Pop_3, respectively. The line "Pool" in the "Chromosome" column corresponds to the pooled analysis of the 10 A chromosomes together. Column details: 'Pop_n' refers to the name of the hybrid n, 'KL_n' gives the KL

divergence value between the observed distribution of crossovers for the hybrid n, and the corresponding flat histogram (H0 hypothesis), ‘pvalH0_n’ corresponds to that H0 hypothesis, and ‘pvalDiffKL_n-p’ refers to the p-value of the hypothesis “KL_n - KL_p=0”.

Table S3. Landscapes comparison analysis. Detail of the KL values and p-values corresponding to the results presented in Figures 3C and 6B. The numbers 1, 2, 3 indicate the hybrids of the columns Pop_1, Pop_2 and Pop_3, respectively. The line "Pool" in the "Chromosome" column corresponds to the pooled analysis of the 10 A chromosomes together. Column details: ‘Pop_n’ refers to the name of the hybrid n. ‘KL_n-p’ gives the values of the KL divergences between observed distributions of crossovers in hybrids of Pop_n and Pop_p. ‘Inf_n-p’ and ‘Sup_n-p’ indicate the corresponding confidence intervals at α=5% (2.5% on each side, from 10^4 bootstraps).

Table S4. Chi-squared comparisons between hybrids carrying *B. oleracea* C_o or *B. napus* C_n chromosomes for crossover rate heterogeneity per interval between adjacent linked SNP markers.

Table S5. Genetic distances in centimorgan (cM) between linked SNP markers for each hybrid. Intervals including pericentromeric regions are indicated in grey.

Table S6. Interference analysis. Detail of the KL values and p-values corresponding to the results presented in Figure 5 and Supplementary Figures S6 and S7. The numbers 1, 2, 3 indicate the hybrids of the columns Pop_1, Pop_2 and Pop_3, respectively. The line "Pool" in the "Chromosome" column corresponds to the pooled analysis of the 10 A chromosomes together. Column details: ‘Pop_n’ refers to the name of the hybrid, ‘KL_n’ gives the value of the KL divergence between the observed distribution of distances between successive crossovers for the hybrid n, and the corresponding distribution without interference obtained by shuffling the data (H0 hypothesis), ‘pvalH0_n’ corresponds to that H0 hypothesis, and ‘pvalDiffKL_n-p’ refers to the p-value of the hypothesis “KL_n – KL_p=0”.

Table S7. Meiotic behavior observed in Pollen Mother Cells at Metaphase I of hybrids carrying combinations of C_n chromosomes from *B. napus*. % Cells: percentage of cells with the expected behavior. I and II represent univalents and bivalents, respectively.

Table S8. Comparison of the meiotic gene content on the C09 chromosome between *B. napus* cv. ‘Darmor’ and *B. oleracea* cv. ‘RC34’.

Table S9. List of the meiotic gene content on the C04 and C08 chromosomes of *B. napus* cv. 'Darmor' and their orthologous genes in *A. thaliana*.

Table S10. Characteristics of SSR markers used to study the segregation of *B. napus* C_n chromosomes.
