# Supplementary figures and images for: Genomic Divergence Shaped the Genetic Regulation of Meiotic Homologous Recombination in Brassica Allopolyploids
Source: Mol Biol Evol. 2025 Apr 2;42(4):msaf073. doi: 10.1093/molbev/msaf073 (PMC11982612; doi:10.1093/molbev/msaf073)

**A**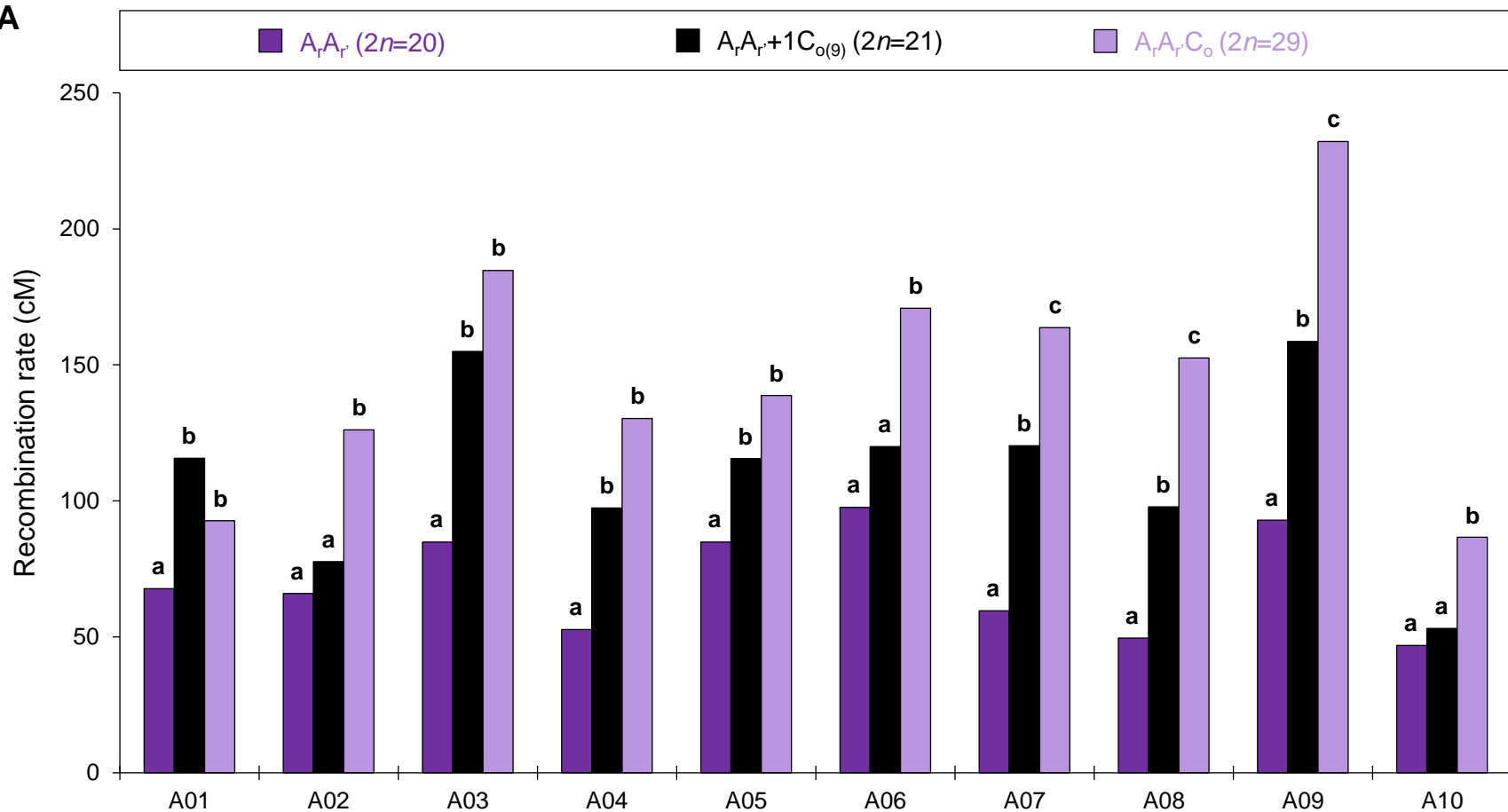**B**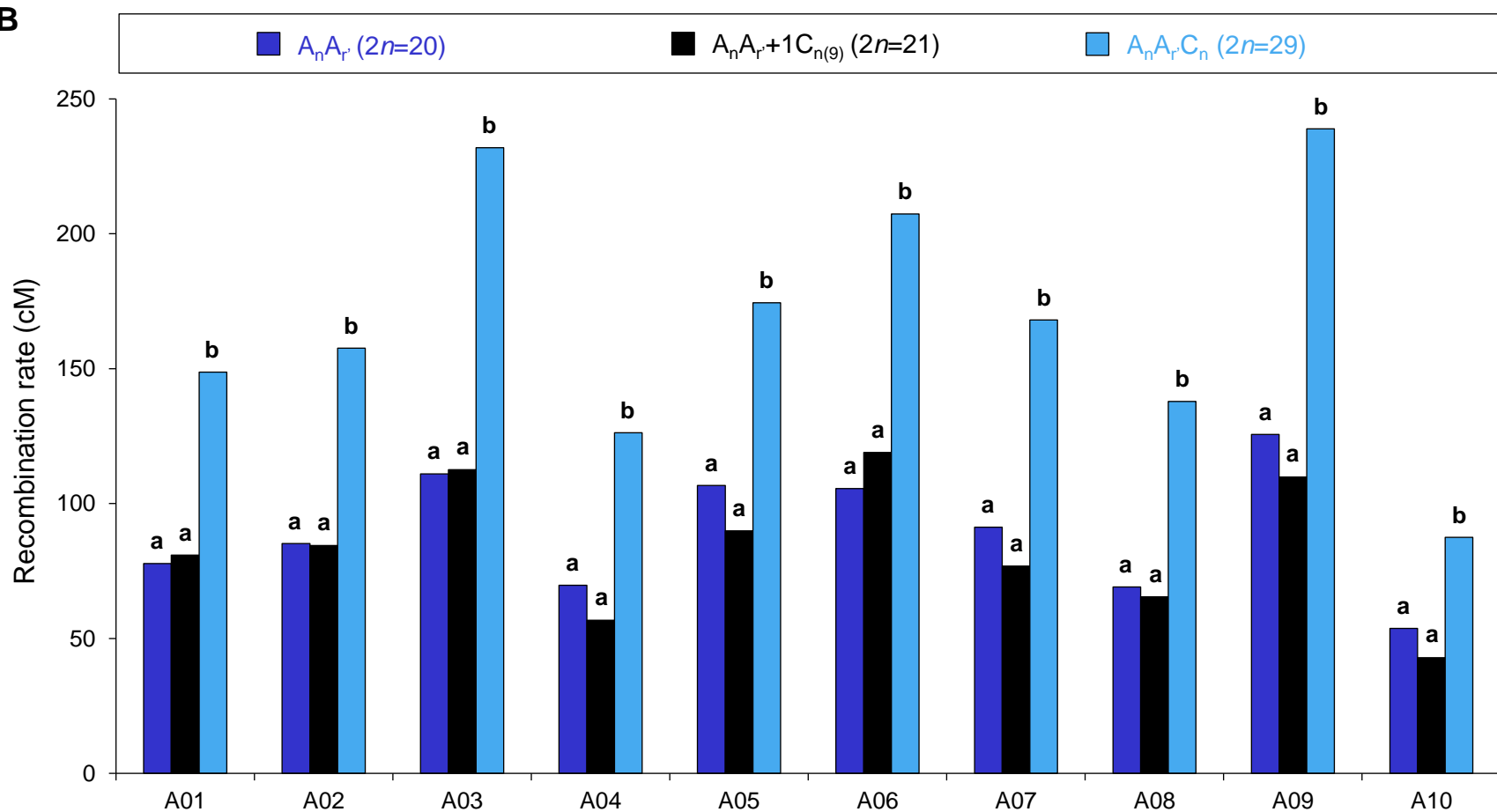

Supplement: msaf073_Supplementary_Data [file msaf073_supplementary_data.zip › Fig. S1.pdf]

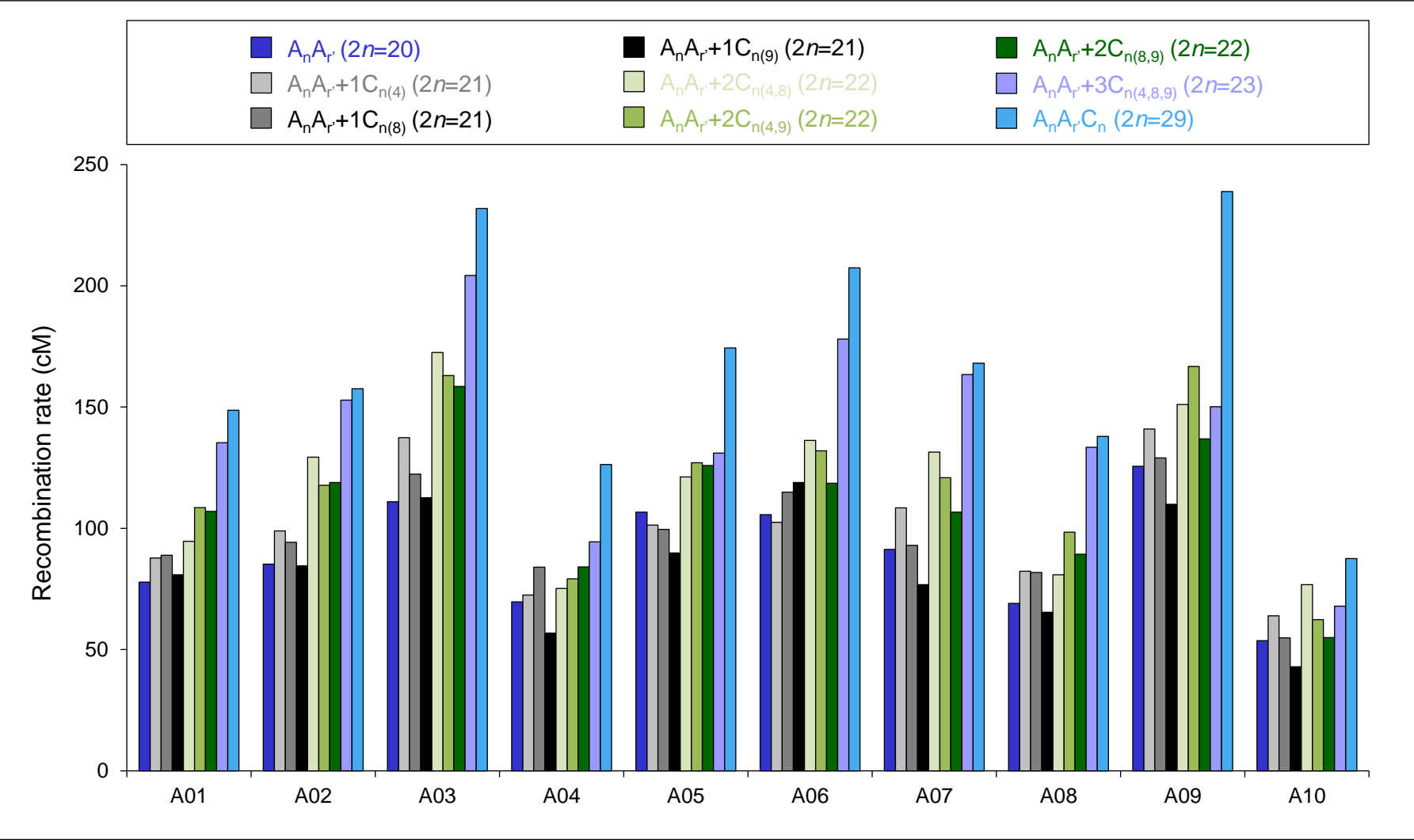

Supplement: msaf073_Supplementary_Data [file msaf073_supplementary_data.zip › Fig. S10.pdf]

$A_rA_r$  ( $2n=20$ )

$A_rA_r+1C_{o(9)}$  ( $2n=21$ )

$A_rA_rC_o$  ( $2n=29$ )

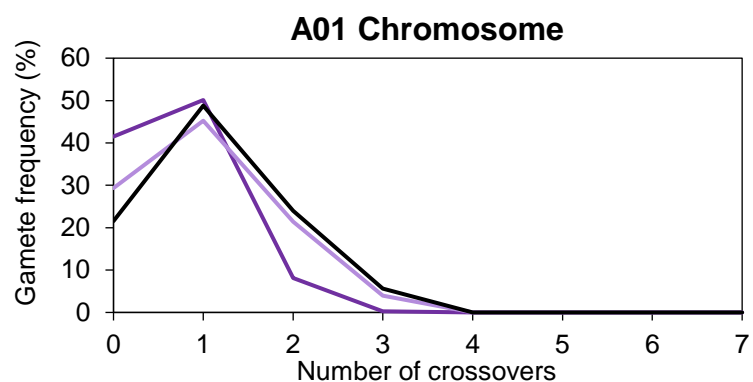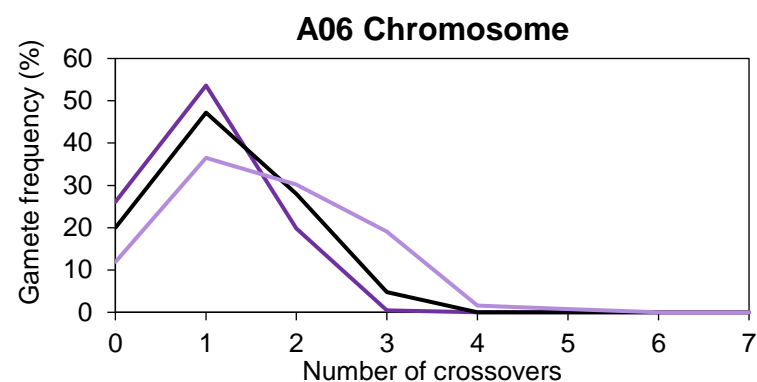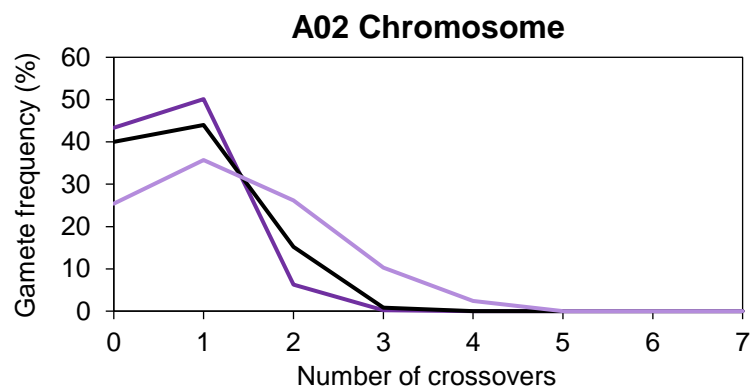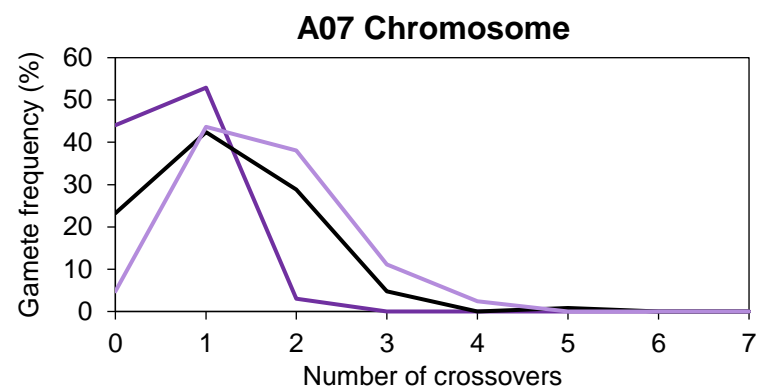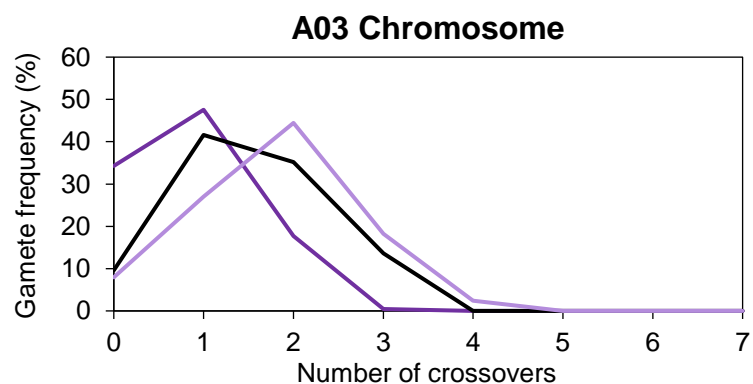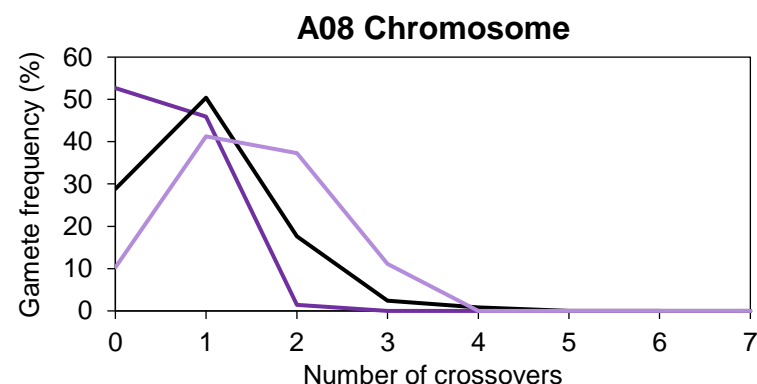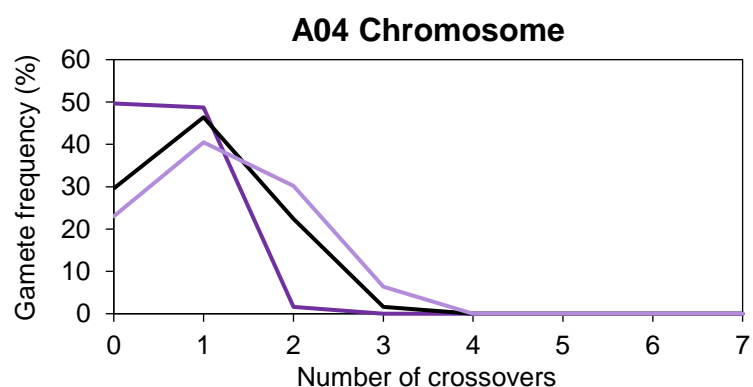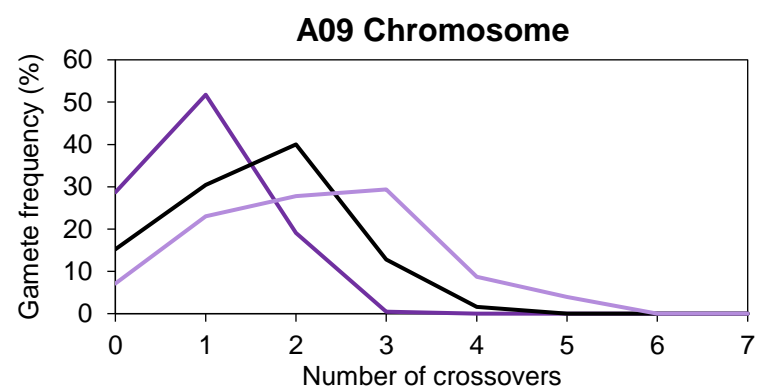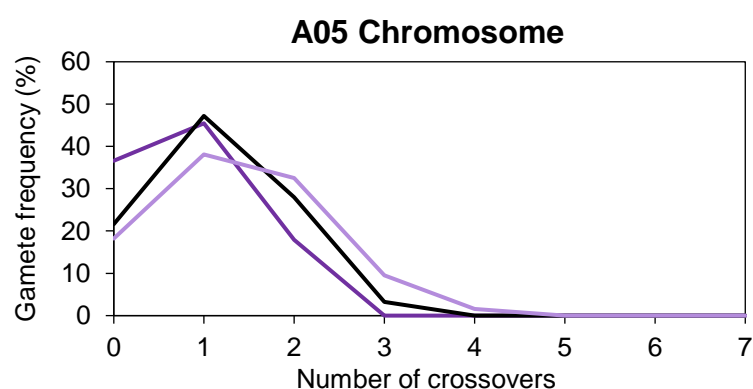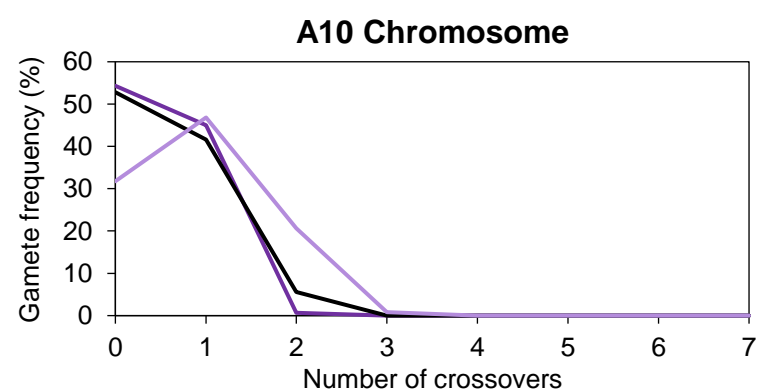

Supplement: msaf073_Supplementary_Data [file msaf073_supplementary_data.zip › Fig. S2.pdf]

**A**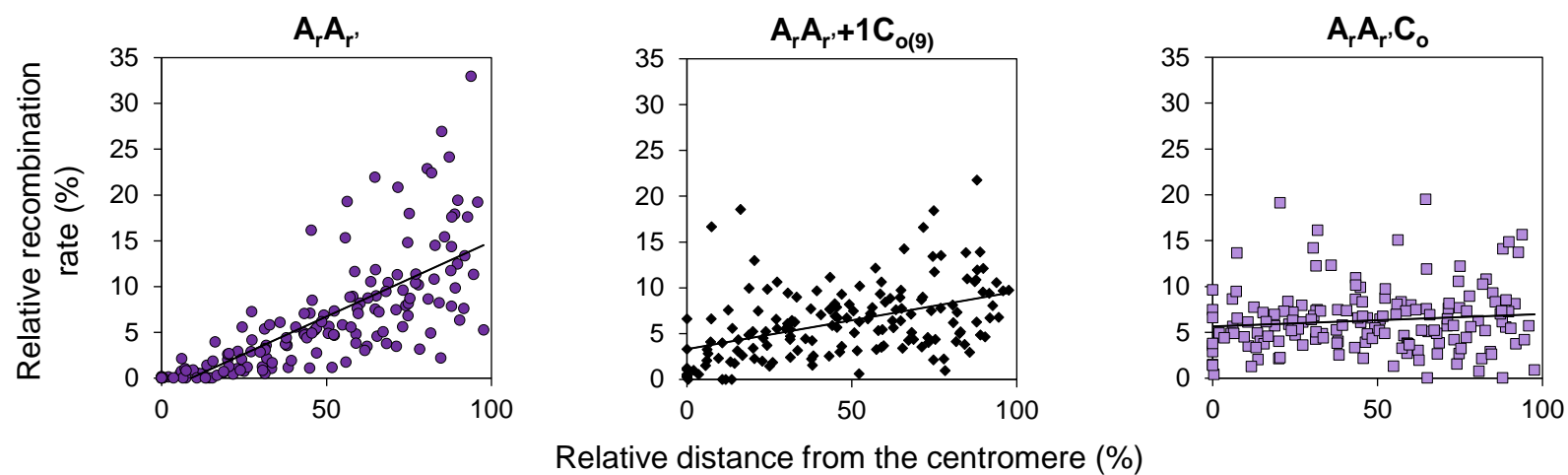**B**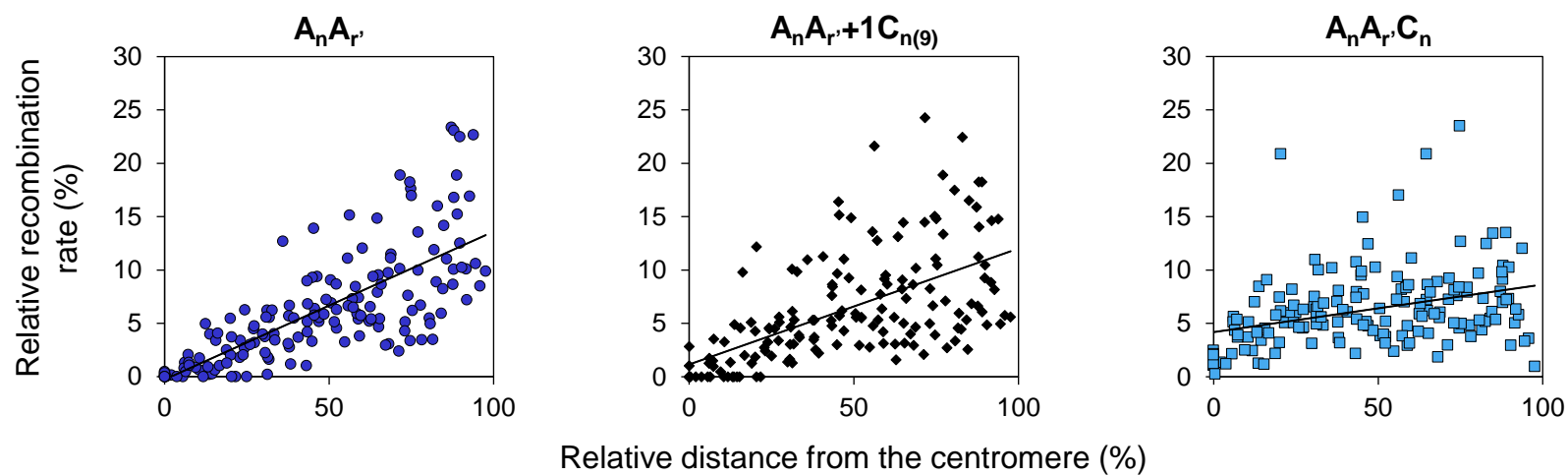

Supplement: msaf073_Supplementary_Data [file msaf073_supplementary_data.zip › Fig. S5.pdf]

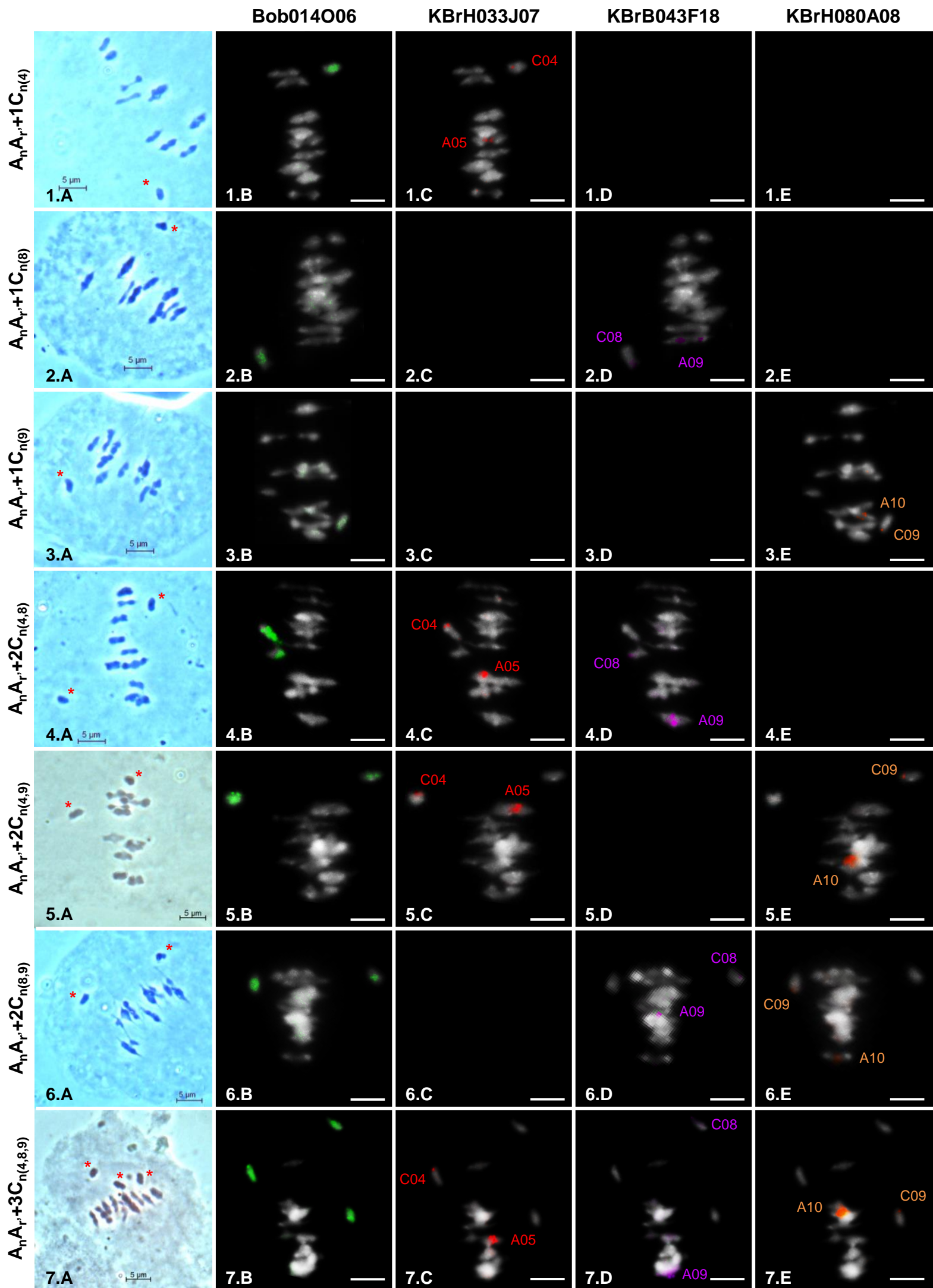

Supplement: msaf073_Supplementary_Data [file msaf073_supplementary_data.zip › Fig. S8.pdf]

$A_nA_r$  ( $2n=20$ )

$A_nA_r+1C_{n(9)}$  ( $2n=21$ )

$A_nA_rC_n$  ( $2n=29$ )

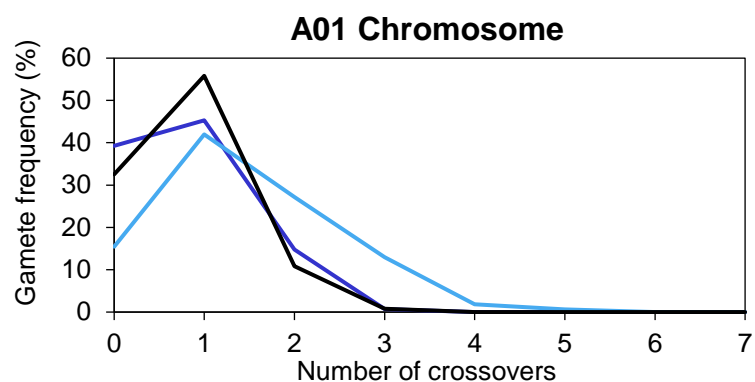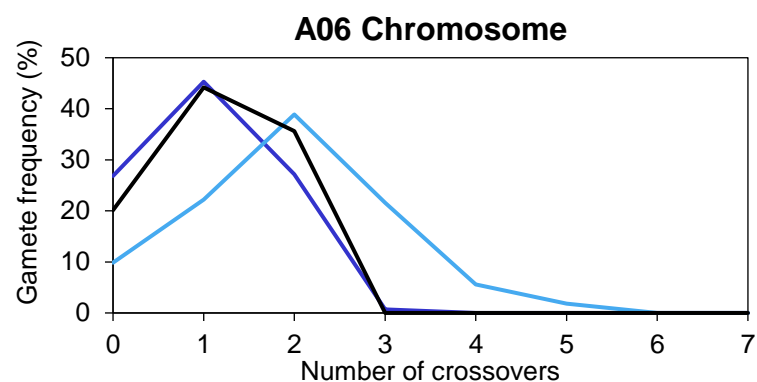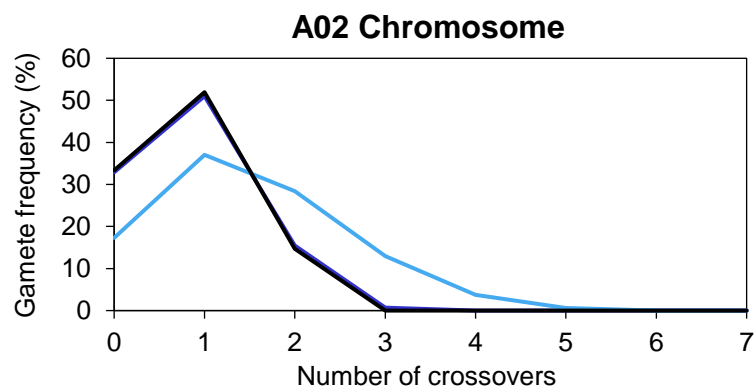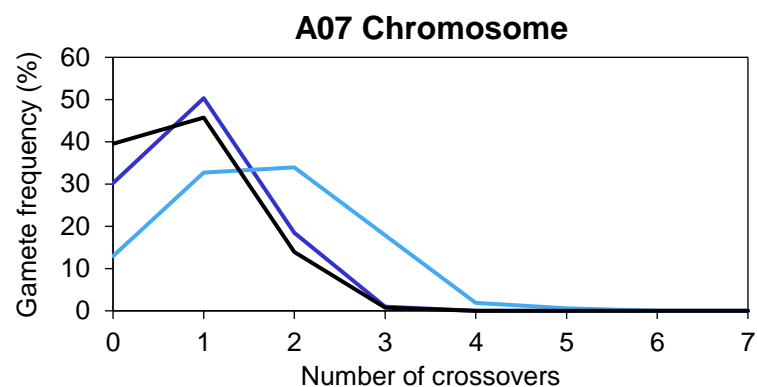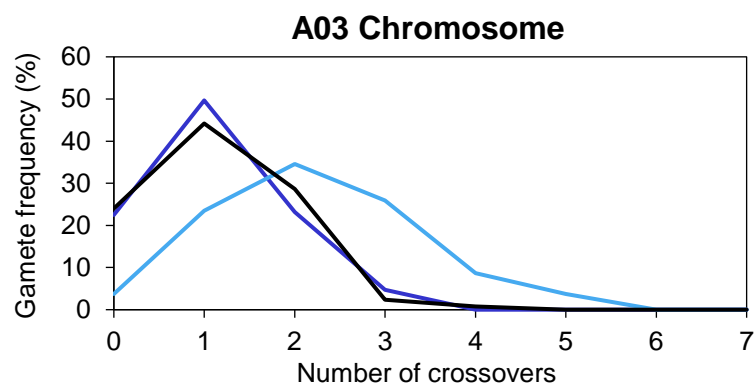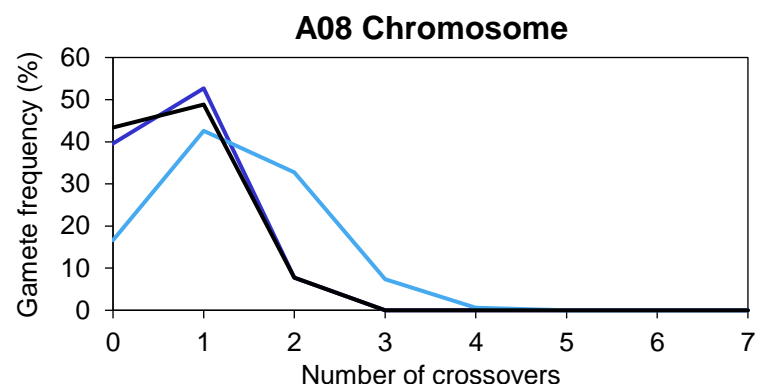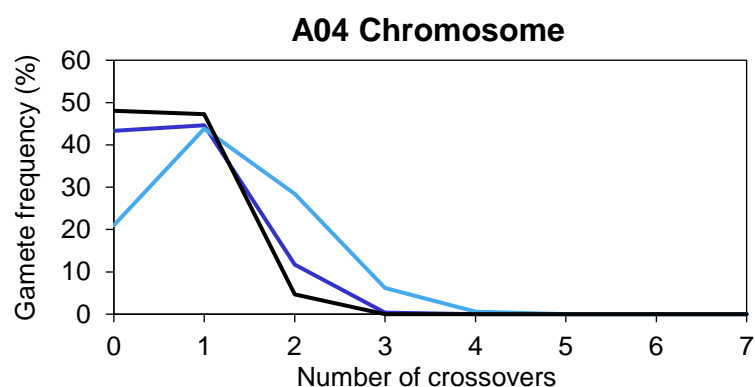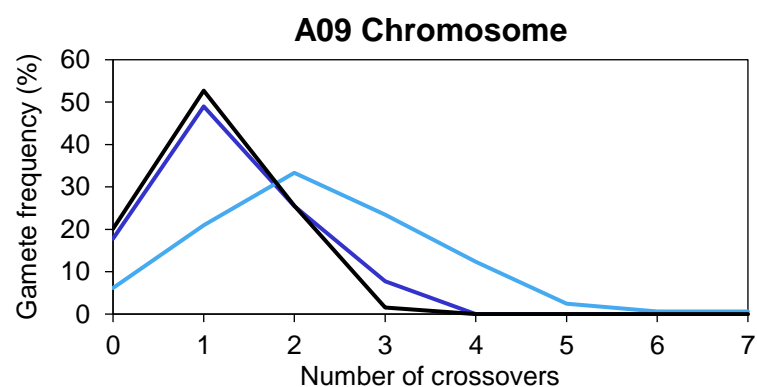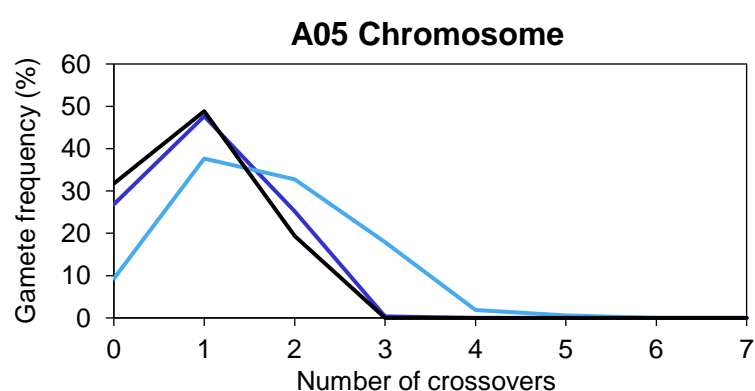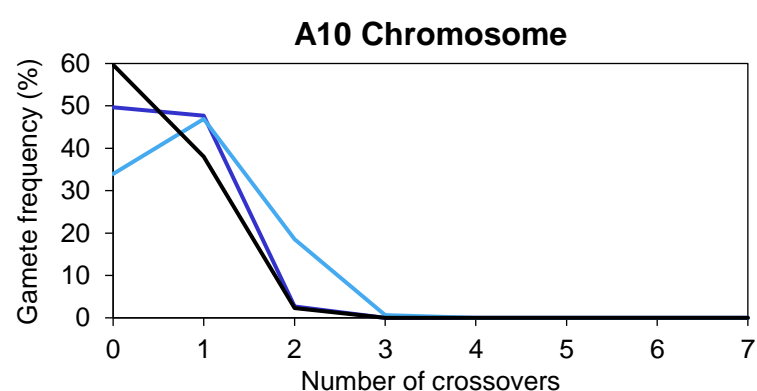

Supplement: msaf073_Supplementary_Data [file msaf073_supplementary_data.zip › Fig. S9.pdf]
